# An Evaluation of the National Brucellosis Surveillance System in Qatar, 2018

**DOI:** 10.7759/cureus.4169

**Published:** 2019-03-03

**Authors:** Ayatullah A Mohamed, Mohamad A Chehab, Ayman Al-Dahshan, Hamad E Al-Romaihi, Elmoubasher A Farag

**Affiliations:** 1 Community Medicine, Hamad Medical Corporation, Doha, QAT; 2 Preventive Medicine, Hamad Medical Corporation, Doha, QAT; 3 Epidemiology and Public Health, Ministry of Public Health, Doha, QAT

**Keywords:** brucella, surveillance, evaluation, qatar

## Abstract

Introduction

Brucellosis is one of the most prevalent bacterial zoonoses and is considered an economically important infection that affects humans and livestock. The infection is usually transmitted to humans through direct contact with infected materials, such as the afterbirth, or indirectly through the ingestion of animal products. In addition, the consumption of raw milk represents a major source of the infection. In the Eastern Mediterranean region, the incidence of brucellosis ranges from one per 100,000 to 20 per 100,000; however, the actual figure is estimated at 20 to 25 times greater owing to the poor surveillance systems among countries in the region. For such reasons, this study is conducted to comprehensively evaluate the brucellosis surveillance system in Qatar, to identify potential strengths and limitations and, hence, inform decision-makers about future mitigation strategies.

Methods

A retrospective record review was conducted at the surveillance unit in the Ministry of Public Health (MoPH) to analyze all Brucella notification forms from January to November 2018 for the completeness of notification and timeliness of reporting. The principal investigators conducted data abstraction and analysis in November 2018.

Results

A total of 125 notification forms were analyzed. It was revealed that the internal completeness varied across the different data elements of the notification forms from 39% up to 100%. Also, the timeliness of the reporting ranged from one day for the T3 time point up to 16 days for the T1 time point.

Conclusion

Ultimately, the strengthening of the national Brucellosis surveillance system in Qatar demands the implementation of several strategies, including the establishment of veterinary surveillance, enforcement of livestock importation protocols, as well as routine compulsory vaccinations, devising a clear and sensitive case definition of the disease, and public education especially among high-risk groups (shepherds, slaughterhouse workers, and laboratory workers). In addition, continuous education of healthcare workers on the proper reporting of the disease and the electronization of the notification process are important steps to improve the surveillance system in the country.

## Introduction

The effective control of any communicable disease relies on a well-established surveillance system for such disease. Similarly, a functional national communicable diseases surveillance system is essential for any effective action on priority communicable diseases. Moreover, a surveillance system constitutes a key part of the public health decision-making across all countries (e.g. priority setting, planning, resource mobilization and allocation, prediction and early detection of epidemics, and monitoring and evaluation of disease prevention and control programs) [1].

The interface between human beings, animals, and the environment can be a source of many zoonotic diseases with adverse implications on the public health system as well as the social and economic aspects [2].

Brucellosis is one of the most prevalent bacterial zoonoses and is considered an economically important infection that affects humans and livestock. The infection is usually transmitted to humans through direct contact with infected materials such as the afterbirth or indirectly through the ingestion of animal products. In addition to that, the consumption of raw milk represents a major source of the infection [3]. The annual incidence of the disease reaches more than 500,000 cases worldwide. Nevertheless, the distribution of brucellosis varies widely across regions, where it has been eliminated in a number of high-income countries such as Australia, Canada, Japan, New Zealand, and some European countries. On the other hand, it remains a major public health concern in the Eastern Mediterranean Region (EMR), African Region, Region of the Americas, and parts of Asia [4].

In the EMR, the incidence of brucellosis ranges from one per 100,000 to 20 per 100,000; however, the actual figure is estimated at 20 to 25 times greater, owing to the poor surveillance systems among countries in the region [5]. The highest incidence of brucellosis arose from Syria, Lebanon, Iraq, Saudi Arabia, Sudan, and Oman [6]. Despite such a high burden of brucellosis in many developing countries, the disease is often neglected. Therefore, the World Health Organization (WHO) is currently classifying brucellosis as one of the top neglected zoonoses worldwide [7].

Regarding the situation in Qatar, brucellosis is a non-endemic disease and shows a low incidence when compared to other countries in the EMR region. The incidence rate of brucellosis has been declining between 2004 and 2012, with the highest figure being in 2006 (4.2 cases per 100,000 inhabitants). However, the risk of brucellosis outbreaks is probable, as the prevalence of raw milk consumption and direct animal contact in Qatar are reported at 41.7% and 12.5%, respectively. Also, the country has recently witnessed a huge rise in production of dairy products, with the importation of significant number of livestock for such a reason [8]. Furthermore, an earlier outbreak of human brucellosis among 14 family members in Qatar was reported and further investigation has identified the consumption of infected raw camel milk to be the source [9]. Thus, this study has been conducted to comprehensively evaluate the brucellosis surveillance system in Qatar, to identify potential strengths and limitations, and hence inform decision-makers for future mitigation strategies.

## Materials and methods

Setting and sampling 

Qatar’s national surveillance system categorizes notifiable diseases into two classes depending on national and international disease-reporting regulations. Diseases that are immediately notifiable (within 24 hours) through telephone or fax are denoted as Class A diseases while those that may be notified as soon as possible (up to one week) are designated as Class B diseases. Additionally, standard definitions of case reporting have been developed, along with guidelines and standard operating procedures, for disease surveillance, reporting, and control. As of 2018, the list of notifiable diseases in the State of Qatar encompassed 78 diseases, including brucellosis, which is classified as a Class B disease. 

A retrospective record review was conducted at the surveillance unit in the Ministry of Public Health (MoPH) to analyze all Brucella notification forms during the year 2018 for the completeness of notification and timeliness of reporting. The principal investigators conducted data abstraction and analysis in November 2018. A total of 125 notification forms were analyzed.

Data collection

The first attribute of the surveillance system, completeness, was evaluated from internal aspects. Regarding internal completeness, a data extraction sheet was established based on the minimum data elements to be filled in the notification form as recommended by the WHO, such as the number of cases by case classification (suspected/confirmed), unique identifier, age, sex, geographical information, and occupation [1]. The aforementioned data elements were evaluated based on their availability and legibility. Subsequently, an element was considered available if it was written in the form and considered legible if it was clear as well as readable. Thus, the principal investigators evaluated all the available forms and a variable was judged legible if it was clear and readable by the researchers.

In addition to that, the timeliness attribute of the surveillance system was evaluated by calculating the time lag between the key time points of the Brucella surveillance system: date of symptoms’ onset, date of diagnosis, and date of notification receipt by the central surveillance unit. Thus, three key time points were considered T1 (timeliness between the onset of symptoms and the receipt of notification at MoPH), T2 (timeliness between the onset of symptoms and diagnosis), and T3 (timeliness between disease reporting and receipt of notification). The reporting sites were classified into five categories: Hamad Medical Corporation (HMC) facilities (Hamad General Hospital-HGH, Al Khor Hospital-AKH, Al Wakra Hospital-AWH, and The Cuban Hospital-CH), Primary Health Care Corporation (PHCC) health centers, Qatar Red Crescent (QRC), the private sector, and others. Moreover, HMC is the main government-based provider of hospital care for the residents in the State of Qatar. Also, the PHCC is a governmental provider of primary health care (and some secondary health care) through 27 health care centers dispersed across Qatar.

Data analysis

A descriptive analysis of the notification forms was performed using Microsoft Excel 2016. The proportions of complete data elements in the notification forms and the timeliness of notification were calculated.

## Results

**Figure 1:**
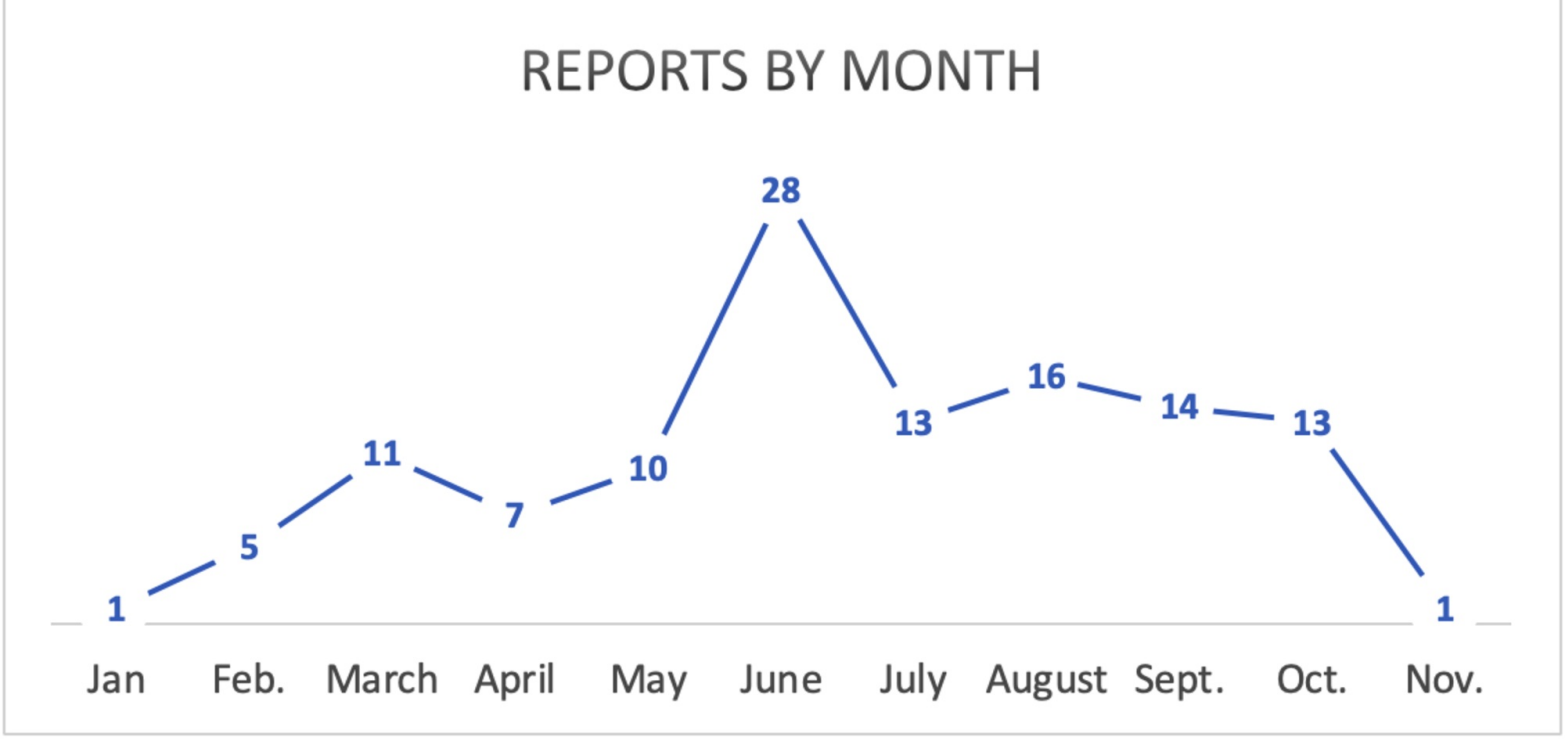
Brucella reports notifications by month, Qatar, 2018

Between the months of January and November 2018, a total of 125 new Brucella cases were notified. Almost a third (32.8%) of the cases were reported through the laboratory-based notification system. During the same interval, less than two-thirds (63.2%) of the received Brucella notifications were from the central surveillance unit were from physicians while a negligible percentage (4%) were missing the source. The bulk of notified Brucella cases were registered during the months of June, July, August, and September while January and November witnessed the lowest number of notifications (Figure 1).

Similarly, the frequency of diagnosed and reported cases shared a similar trend over time. Regarding the contribution of each institution to the overall notification process, the central laboratory under the HMC and HMC hospitals ranked first after accounting for almost two-thirds of the notification forms when combined (33.8% and 30.5%, respectively). The reporting forms from the private sector, as well as QRC, contributed up to one-sixth (16.1%) of the total notifications received. Additionally, the least reporting site was the PHCC (10.7%).

Furthermore, nine of the data elements in the notification forms were analyzed for internal completeness and included the age, gender, nationality, address, travel history, occupation, contact with animals or their products, unique identification, and diagnosis. The unique identifier was the most frequently reported data field and found to be reported in 100% of the notifications. Similarly, the age and gender data elements were available in the majority (>91%) of the notification forms. However, the least reported data components were occupation, contact information, address, and travel destination with the following percentages of 63%, 47%, 39%, and 49%, respectively. While the internal completeness of the date of diagnosis and nationality valued at 92% and 80%, respectively. 

Regarding the timeliness of reporting, the overall median time lag was 16 days for T1, 14 days for T2, and one day for T3, associated with a broad range of two to 114 days, zero to 113 days, and zero to 24 days for T1, T2, and T3, respectively.

## Discussion

In the current study, two attributes of the national Brucella surveillance system in Qatar were evaluated. It was revealed that the internal completeness varied across the different data elements of the notification forms, from 39% up to 100%. Also, the timeliness of the reporting ranged from one day for the T3 time point up to 16 days for the T1 time point.

Most cases of brucellosis were reported during the summer season, which correlates well with the established seasonal variation of the disease incidence that usually peaks during the summer. During the aforementioned season, there is increased exposure to Brucella due to the occurrence of births and abortions among cattle, camels, and sheep as well as the consumption of raw milk [10]. An earlier study on brucellosis in Qatar has revealed that raw milk consumption was quite prevalent (41.7%) among the diagnosed cases of brucellosis in a hospital-based setting [8].

The low contribution (10.7%) of PHCC, the main provider of primary health care in Qatar, to the overall Brucella notification process is concerning, given the vital role of primary health care in the control of brucellosis [10]. In addition to that, primary healthcare professionals must be aware of the symptoms of acute and chronic brucellosis to properly manage the patients or refer them to secondary or tertiary specialized care [11]. Furthermore, primary care physicians and nurses should provide health education to clients at high risk of acquiring brucellosis such as camel workers, farmers, laboratory workers, consumers of raw milk, and others [10].

Although brucellosis is categorized as a Class B notifiable infectious disease in Qatar, the current evaluation has revealed that the median time between the onset of symptoms and the receipt of disease notification by MoPH (T1) reached 16 days; more than double the recommended duration of one week as denoted in the national reporting guidelines. On the other hand, an earlier evaluation of the national malaria surveillance system in Qatar has found the previously mentioned time point to be six days, given the fact that malaria is also a Class B notifiable disease in the country [12]. To optimize the timeliness of reporting infectious diseases, previous research has advocated the implementation of an electronic notification system as well as a laboratory-based surveillance system [13-14].

Recently, the domestic production of dairy products in Qatar has witnessed a substantial growth with the importation of cattle from abroad and the aim to reach a self-sufficient local market [15]. According to the Livestock department at the Ministry of Municipality and Environment, there are approximately 1.5 million livestock in Qatar, including 40,000 cows and 70,000 camels [16]. Thus, to maintain food safety while achieving food security in Qatar, it is vital to ramp up active surveillance through the One Health Approach based on an intersectoral and multidisciplinary collaboration between the various involved ministries and institutions such as the Ministry of Public Health and the Ministry of Municipality and Environment.

## Conclusions

Ultimately, the strengthening of the national brucellosis surveillance system in Qatar demands the implementation of several strategies, including the establishment of veterinary surveillance, enforcement of livestock importation protocols, as well as routine compulsory vaccinations, devising a clear and sensitive case definition of the disease, and public education, especially for high-risk groups (shepherds, slaughterhouse workers, and laboratory workers). In addition, the electronization of the notification process and the continuous education of healthcare workers on the proper reporting of the disease are important steps to improve the surveillance system in the country.
